# Novel fixed-target serial crystallography flip-holder for macromolecular crystallography beamlines at synchrotron radiation sources

**DOI:** 10.1107/S1600577524011664

**Published:** 2025-02-03

**Authors:** Do-Heon Gu, Dong Tak Jeong, Cheolsoo Eo, Pil-Won Seo, Jeong-Sun Kim, Suk-Youl Park

**Affiliations:** a Department of Chemistry Chonnam National University Gwangju Republic of Korea; b Pohang Accelerator Laboratory Pohang University of Science and Technology Pohang Republic of Korea; RIKEN SPring-8 Center, Japan

**Keywords:** serial crystallography, room temperature, synchrotron, flip-holder, fixed-target holder

## Abstract

Synchrotron serial crystallography (SSX) is an emerging method for determining crystal structure at room temperature using synchrotron radiation facilities. Despite the various approaches available, reducing sample consumption, removing mother liquid from crystal solution, soaking small molecules for protein–ligand complex structure and prevention of sample dehydration are still challenging problems to be overcome in SSX. Therefore, we have developed a new flip-type fixed-target SSX sample holder for conventional protein crystallography beamlines based on nylon mesh and kapton film. The potential of the flip-holder was evaluated in an SSX experiment with lysozyme crystals at room temperature. About 19600 diffraction images were collected during 40 minutes using a repetition rate of 10 Hz at the 11C beamline of the Pohang Accelerator Laboratory, and the crystal structure of lysozyme was determined at 1.89 Å resolution. This straightforward flip-holder can be used in synchrotron beamlines for routine crystallography.

## Abstract 2

We present the development of a novel fixed-target flip-holder for synchrotron serial crystallography. This holder simplifies the sample handling process, reduces dehydration risks, and enables efficient crystallography experiments with minimal sample consumption, demonstrated through successful lysozyme crystal structure determination at room temperature.

## 1. Introduction

X-ray crystallography has been a powerful method for determining protein structure at the atomic level (Watson & Crick, 1953 ▸; Anfinsen, 1973 ▸; Brockhouse & Shull, 1995 ▸; Shukla *et al.*, 2013 ▸). However, conventional crystallography is carried out at cryogenic temperatures to minimize X-ray induced damage. Serial crystallography (SX) enables the observation of structures at room temperature, which is close to physiological structure, although the molecules are packed in a crystalline system (Chapman *et al.*, 2011 ▸; Nogly *et al.*, 2015 ▸; Weinert *et al.*, 2017 ▸; Kim *et al.*, 2018 ▸). In comparison with the single crystals used in conventional crystallography, SX data collections are performed with thousands of crystals at room temperature. Thus, SX merges tens of thousands of partial diffraction images from diversely oriented microcrystals. SX is a process of introducing multiple crystals to the beam. Injector-based SX requires mixing the sample with a delivery medium (such as lipidic cubic phase) (Grünbein & Nass Kovacs, 2019 ▸; Park & Nam, 2019 ▸). On the other hand, fixed-target SX needs a crystal sample holder to position crystals on the beamline goniometer (Hunter *et al.*, 2014 ▸). In fixed-target SX, the X-ray beam hits crystals on a moving sample holder. The fixed-target method has the major advantage of using a smaller amount of crystal sample than the injector-based method for sufficient data collection and the used crystal sample can even be reused for additional diffraction trials (Oghbaey *et al.*, 2016 ▸; Owen *et al.*, 2017 ▸; Shelby *et al.*, 2020 ▸).

Several fixed-target serial crystallography holders including silicon chips, silicon nitride membrane, kapton (polyimide) film holders and graphene chips have been developed (Zarrine-Afsar *et al.*, 2012 ▸; Roedig *et al.*, 2015 ▸; Feld *et al.*, 2015 ▸; Baxter *et al.*, 2016 ▸; Lee *et al.*, 2019 ▸; Frank *et al.*, 2014 ▸; Hunter *et al.*, 2014 ▸; Murray *et al.*, 2015 ▸; Li *et al.*, 2018 ▸). These sample holders with holes of a certain size were advantageous for removing parasitic scattering from the holder for optimal diffraction peak processing and positioning the crystals at discrete positions (allowing for laser pump–probe experiments). However, they are expensive to manufacture and require precise alignment. ‘Sheet-on-sheet’ sandwich structures, which utilizes two Mylar sheets, eliminate the need for precise micromachining and simplify sample handling (Doak *et al.*, 2018 ▸). Even so, this approach lacks precise and discrete positioning of crystals and may cause parasitic scattering due to direct contact between the Mylar sheets and the crystals. An alternative format is a nylon mesh and enclosed film (NAM)-based sample holder used for both synchrotron and XFEL beamlines (Lee *et al.*, 2019 ▸; Park *et al.*, 2020 ▸); however, this still requires a time-consuming assembly process for the complete holder.

We have developed a new fixed-target synchrotron serial crystallography (SSX) holder (Doak *et al.*, 2018 ▸; Lee *et al.*, 2019 ▸). This new flip-type sample holder is assembled with magnets and can be easily detached from the goniometer. This can be reused immediately without extra laborious handling work to replace the mesh and the film on the holder for each experiment. Furthermore, as needed, the crystal solution can be separated from the crystal sample through the mesh pores or even changed into a desired buffer or the chemical conditions can be changed. We have successfully carried out fixed-target SSX experiments with this new holder at the BL11C-PAL using HEWL lysozyme crystals at room temperature. This novel fixed-target flip-holder may be useful not only for protein crystallography but also for organic or inorganic small molecule analysis.

## 2. Materials and methods

### 2.1. Assembly of the fixed-target SX flip-holder

**Figure 1:**
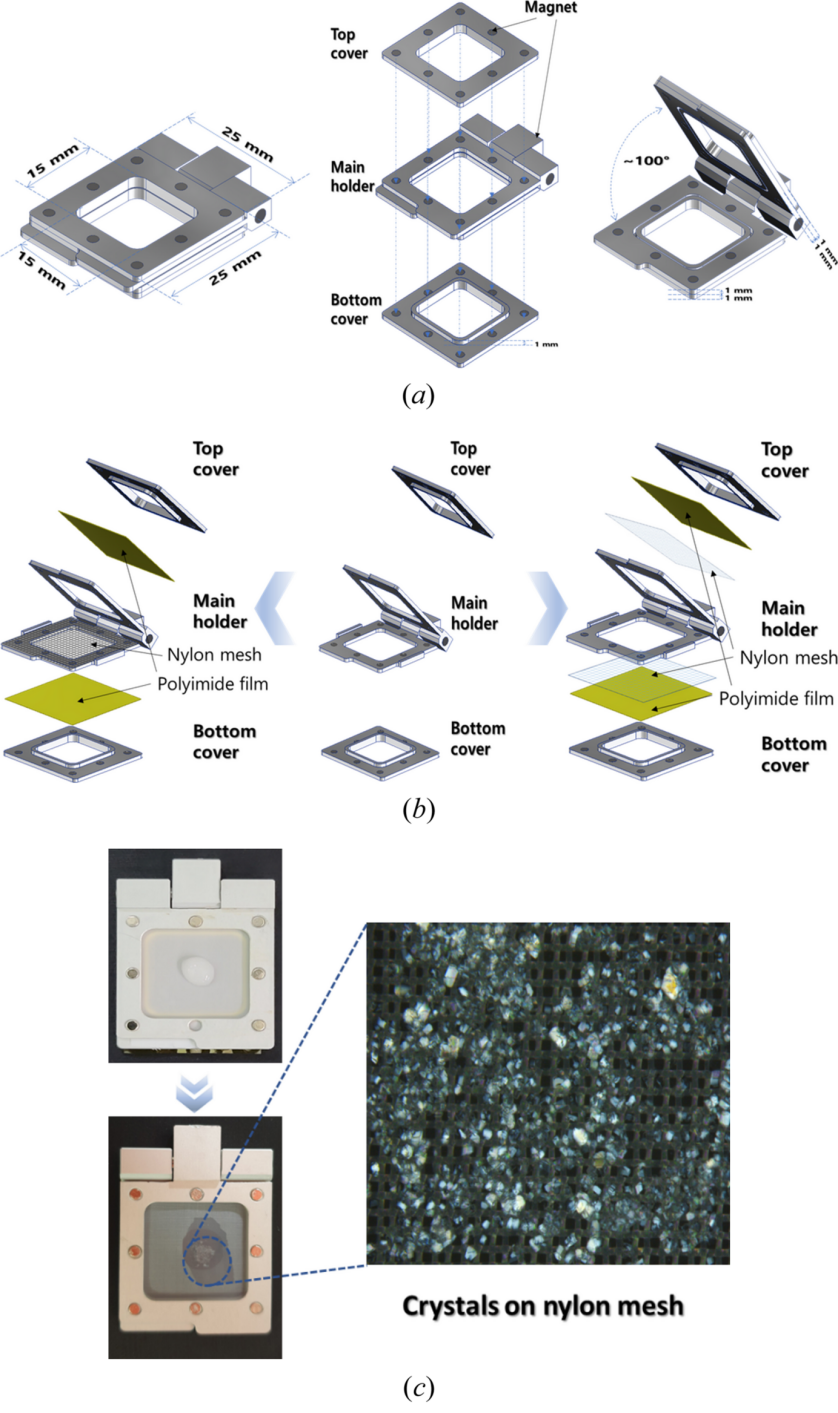
Schematic of the fixed-target holder and photograph of lysozyme crystals on the holder. (*a*) The closed form (left) and the opened form (right) of the holder. Blue dashed lines indicate the length, the opening degree range and the thickness of each part. The separated holder parts are displayed in the center. Blue dashed arrows indicate the magnet attachment system in the holder. (*b*) Two assembly scheme illustrations of the sample holder. One nylon mesh is placed inside the main holder and the kapton films (left). Two nylon meshes and kapton films are placed outside the main holder (right). (*c*) Microscope view of lysozyme crystals loaded on the holder (center). After loading crystal solution on the nylon mesh (top), mother liquid from the crystal solution was removed through opposite side mesh pores by tissue paper (bottom).

The flip-holder consists of an aluminium frame and magnet materials to secure the nylon mesh and polyimide films [Fig. 1 ▸(*a*)].

The flip-holder was assembled with nylon mesh (33 µm pore size and 330 µm thickness) [Fig. 1 ▸(*b*)] for data collection and two kapton films (25 mm × 25 mm). The nylon mesh and kapton films were purchased from Vision Lab Science (Inchon, Republic of Korea) and Covalue Youngjin Co. (Daegu, Republic of Korea), respectively. For a video of the assembly process, see Video S1 of the supporting information. In summary, the top cover, main holder and bottom cover are detached before loading the sample and the trimmed nylon mesh is fixed inside the main holder. The protein crystal solution (30 µL) was dispensed on the nylon mesh and mother liquid was removed from the crystal solution through opposite side mesh pores using tissue paper [Fig. 1 ▸(*c*)]. The weight of the flip-holder, which holds two polyamide films, a nylon mesh and the loaded sample, was 7.0 g when mounted on the goniometer head.

### 2.2. Sample preparation and crystallization

Chicken egg-white lysozyme was purchased from Hampton Research (Cat No. HR7-110; Aliso Viejo, CA, USA). It was dissolved in 20 m*M* sodium acetate pH 4.5 at 100 mg ml^−1^ for crystallization. Crystals suitable for SSX were obtained by batch method. The dissolved lysozyme (60 µL) was mixed with a precipitant solution (200 µL) containing 20 m*M* sodium acetate pH 4.5, 0.9 *M* sodium chloride, and 25%(*v*/*v*) ethyl­ene glycol. Crystals were grown for 12 h at room temperature. The crystal sizes were ∼70 µm (measured by light microscopy).

### 2.3. Data collection

The fixed-target SSX experiments were performed at BL11C-PAL (Park *et al.*, 2017 ▸). Diffraction data were collected at a photon energy of 12.659 keV (0.97942 Å) with a photon flux of 1.3 × 10^12^ photons s^−1^. The X-ray beam was focused to 8.5 µm (horizontal) × 4.1 µm (vertical) (FWHM) at the sample. Data collection was conducted at room temperature (300 ± 1.5 K) and the diffraction images were collected using a PILATUS3 6M detector (Dectris, Baden-Daettwil, Switzerland) at 10 Hz. Raster scans were carried out using an MD2-S X-ray micro-diffractometer (Arinax, Moirans, France). A raster spacing of 50 µm was used with a scanned area of 4.5 mm × 4.5 mm. The crystals were exposed to the X-ray beam for 0.1 s with an oscillation of 1° at each scan point.

### 2.4. Data processing and structure determination

The diffraction images in the total data sets were filtered using *Cheetah* (Barty *et al.*, 2014 ▸). The filtered diffraction images were indexed, integrated, merged and post-refined using *CrystFEL* (White, 2019 ▸). The phasing of lysozyme was processed by molecular replacement using the *Phaser-MR* module in *PHENIX* (Adams *et al.*, 2010 ▸). Iterative model building and computational refinement were performed using *Coot* (Emsley *et al.*, 2010 ▸) and *phenix.refine* in *PHENIX* (Adams *et al.*, 2010 ▸), respectively. Structural figures were prepared using *PyMOL* (available at https://pymol.org/). The data collection and structural refinement statistics are summarized in Table 1 ▸.

## 3. Results

### 3.1. Specifications of the fixed-target SX flip-holder

To enhance the outcomes of SX experiments at synchrotrons, we modified the existing nylon mesh-based sample holder (Lee *et al.*, 2019 ▸). The flip-holder can be quickly mounted onto a goniometer and prevents crystal sample dehydration. When the crystal samples are loaded onto the nylon mesh, the crystal solution flows through the mesh pore to the opposite side. This means that any excess solution can be removed with tissue paper without any physical interaction with the crystal itself. After loading the sample followed by removing the solution and closing the flip-holder, the crystals were fixed in space, allowing them to hold a steady position on the nylon mesh without suffering dehydration. The nylon mesh pore size can be changed depending on the targeted crystal size. The fixed-target SX flip-holder is composed of three parts [Fig. 1 ▸(*a*)]. Each part can be tightly connected by magnetized force and also easily detached. Aluminium was used as the template material and the assembled holder was directly mounted onto a magnet head of the goniometer for X-ray exposure. The entire fixed-target SX holder was designed in a square shape (25 mm × 25 mm) and with an inner square space (15 mm × 15 mm) where the sample is loaded.

### 3.2. Assembly schemes for the fixed-target SX flip-type holder and sample loading

Two different assembly schemes can be applied in the fixed-target flip-holder depending on the position of the nylon mesh within the holder. In the first assembly scheme the nylon mesh is placed into the holder and two kapton films seal the crystals [Fig. 1 ▸(*b*)]. If the crystal size is larger than the nylon mesh pore, the mother liquid from the crystal solution can be easily removed with tissue paper on the opposite side [Fig. 1 ▸(*b*), left]. The kapton films will cover the crystal loading side of the nylon mesh and completely seal the side with the flat state by attaching to the magnetic top cover. The opposite side can be sealed in the same way with the other kapton film and the magnetic bottom cover. The assembled target holder is tightly fixed and can be directly mounted onto the goniometer head without any extra instruments (Video S1). If the crystal sample size is smaller than the nylon mesh pore, or if removing the mother liquid has a negative effect on diffraction, the crystal samples can be sealed between double meshes with a favored solvent. The two nylon meshes and kapton films could be assembled and fixed between the main holder and the top or bottom cover [Fig. 1 ▸(*b*), right]. The double meshes will prevent the loss of crystals which could be flowed out through the mesh pore. This alternative scheme may also be useful for observing binding of small-molecule ligands by injecting the ligand solution into the sample holder after removing the protein crystallizing mother liquid.

### 3.3. Data collection with a fixed-target SSX flip-holder and structural analysis

**Figure 2:**
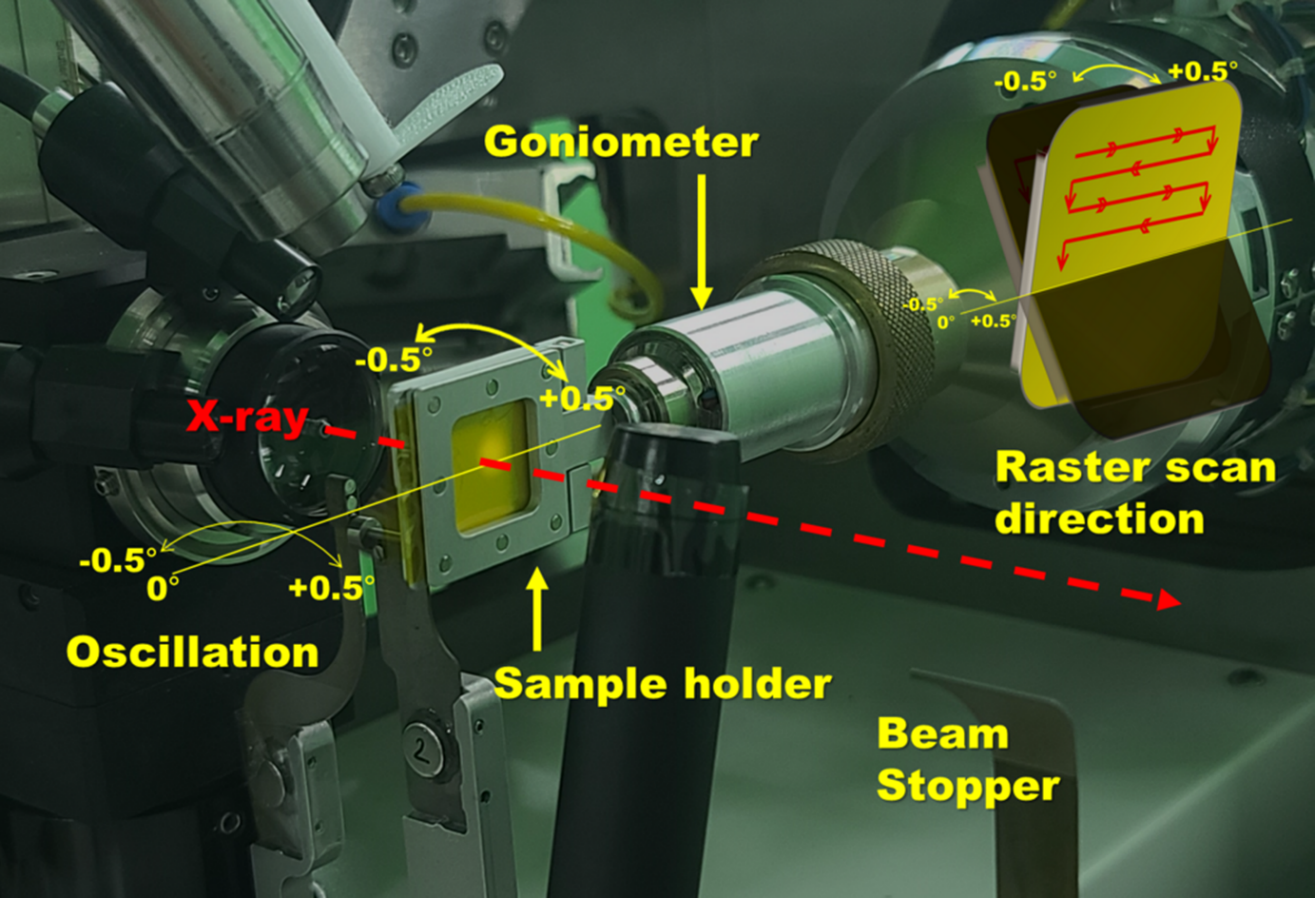
Photograph of the flip-holder mounted on the BL11C beamline (Park *et al.*, 2017 ▸). The X-ray beam is indicated by the red dashed arrow. The sample holder and goniometer are displayed by yellow arrows. The center line of oscillation is displayed by a yellow line. The raster scan direction is displayed by red arrows.

To demonstrate the application of the fixed-target SX holder, an SX experiment was performed using lysozyme crystals as a standard sample. Experimental data were collected at room temperature using the raster scanning method at the BL11C-PAL, where the X-ray beam was focused to 8.5 µm × 4.1 µm (FWHM) (Park *et al.*, 2017 ▸) (Fig. 2 ▸).

A total of 19600 images were collected in 40 minutes. After processing the data using the *Cheetah* package (Barty *et al.*, 2014 ▸), we obtained 5098 useable diffraction images. After optimization of the detector geometry using *geoptimiser* in *CrystFEL*, 3806 images of the total observed images were indexed with a rate of 74.66%. Background scattering from nylon could be observed around 5 Å and 11 Å resolution but was ignored for the purpose of data analysis (see Fig. S1 of the supporting information). Post-refinement was conducted using *partialator* in *CrystFEL*, with the resulting dataset extending to 1.89 Å resolution. The overall signal-to-noise ratio and completeness were 4.8 and 100%, respectively. Overall *R*_split_ and CC* were 16.65% and 0.99, respectively. The average diffraction weighted dose, calculated by *RADDOS-3D* (Zeldin *et al.*, 2013 ▸), was 21.14 kGy.

**Figure 3:**
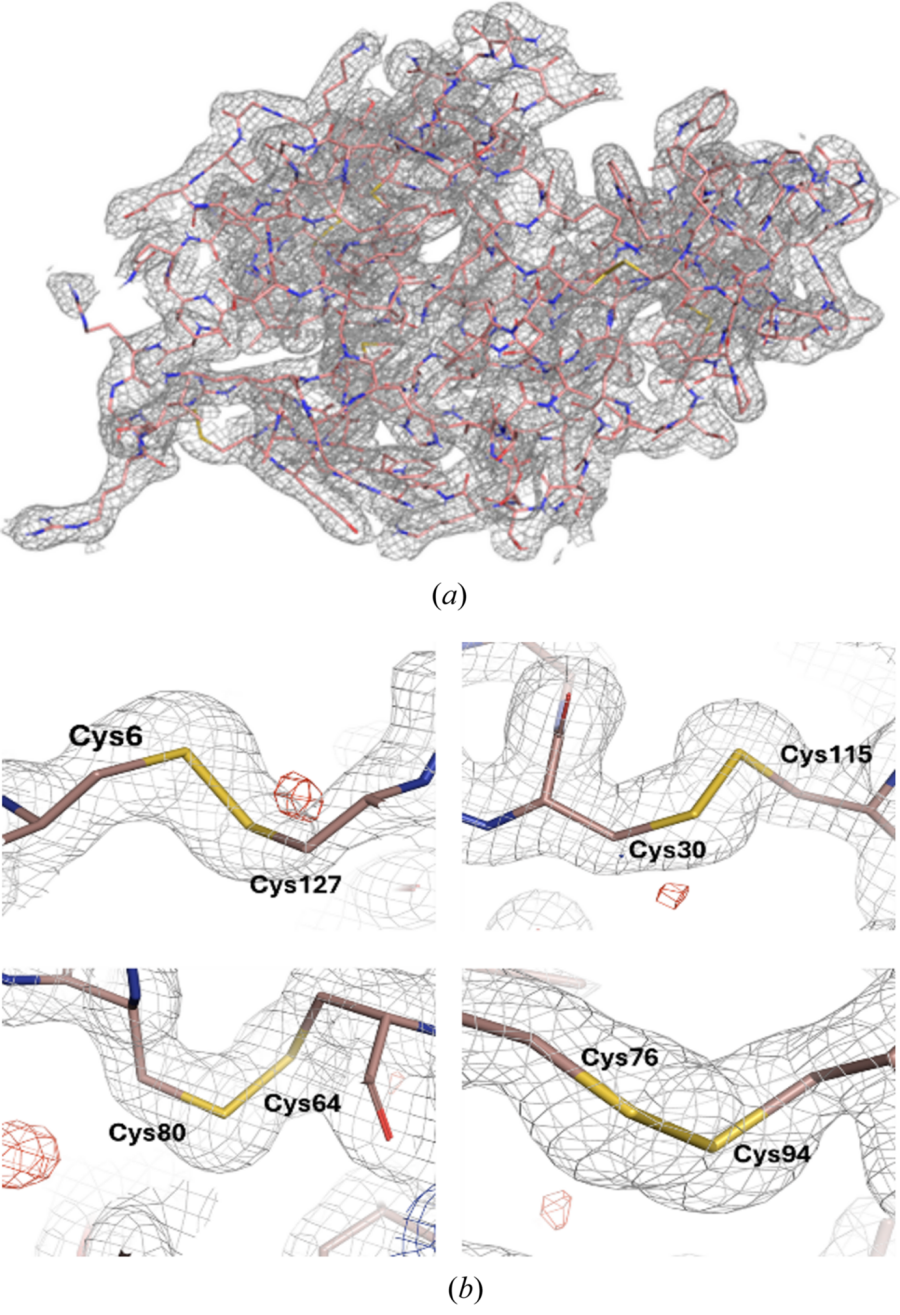
Electron density map and refined structure of lysozyme. (*a*) *2mF*_o_ − *F*_c_ electron density map of lysozyme. The electron density map is presented by a gray mesh at 1.0σ and the lysozyme structure is shown as a stick model. (*b*) *2mF*_o_ − *F*_c_ electron density map (gray) and *mF*_o_ − *F*_c_ electron density map (positive and negative are colored blue and red, respectively) of four different di­sulfide bridges of lysozyme.

*Phaser-MR* in *PHENIX* was used with a starting molecular replacement model (PDB ID, 1vdx) for the lysozyme structure determination. The final model was refined to 1.89 Å, with *R*_work_ and *R*_free_ of 22.81% and 24.57%, respectively. The obtained lysozyme structure [Fig. 3 ▸(*a*)] using our fixed-target holder showed high similarity with the lysozyme structures obtained at room temperature using other methods, gas dynamic virtual nozzle (PDB ID, 4et8) and a droplet injector (PDB ID, 5dm9) with a maximum r.m.s. deviation of 0.232 Å for all Cα atoms. Within the obtained lysozyme structure, four di­sulfide bonds (Cys6—Cys127, Cys30—Cys115, Cys64—Cys80 and Cys76—Cys94) which can be broken by radiation (Contreras-Montoya *et al.*, 2019 ▸) were found to show no characteristic signs of radiation damage [Fig. 3 ▸(*b*)].

## 4. Discussion

An SSX experiment using a novel fixed-target holder was successfully performed at the BL11C-PAL beamline. Room-temperature data collection is a key application of SSX. This novel holder combines inherent design advantages with flexibility, accommodating a diverse range of sample sizes and offering accessibility, making it a practical and versatile tool for room-temperature SSX experiments. Notably, the holder is compatible with conventional macromolecular crystallography beamline setups, eliminating the need for specialized translation axes or complex equipment typically required for *in situ* plate diffractometry.

Recently, two pre-developed SX holders including the kapton film based holder (Li *et al.*, 2018 ▸) and the NAM sample holder (Lee *et al.*, 2019 ▸) were reported with lysozyme structure determination for fixed-target SSX (Table 1 ▸). The new SSX holder based on the NAM has several improvements. Firstly, the SSX sample can be loaded and assembled easily without time-consuming steps. Secondly, the nylon mesh is changeable according to crystal size and simply fixed with magnets. Thirdly, during the sample loading step, the mother liquid of the crystal solution can be easily removed from the crystals or even replaced with a desired buffer or ligand solutions. Lastly, this holder is mounted on the goniometer head by magnets.

Using this flip-holder, we demonstrated an efficient fixed-target approach that significantly reduces both sample consumption and time requirements. By utilizing a small scanning area and an optimized beamline setup, we successfully collected a complete dataset with only 30 µL of sample and approximately 40 minutes of experimental time in a single trial. The observed lower statistics are likely attributable to differences in sample volume, scanning area and crystal density, and could be improved by using more concentrated samples or by increasing the scanned area.

## 5. Conclusions

This new straightforward fixed-target SSX sample holder can be used in general macromolecular crystallography beamlines at most synchrotron radiation sources. Future advancements will improve the efficiency of the SSX experiments by increasing the maximum rotation angle achievable while reducing sample consumption.

## Supplementary Material

Video S1: Assembly video of the novel fixed target serial crystallography holder. One nylon mesh, two polyimide films and lysozyme crystals were used for the sample holder assembly. DOI: 10.1107/S1600577524011664/yi5165sup1.mp4

Figure S1: diffraction images of the nylon mesh and lysozyme crystal. DOI: 10.1107/S1600577524011664/yi5165sup2.pdf

## Figures and Tables

**Table 1:** Data collection, scaling and structure refinement statistics Values in parentheses are for the highest-resolution shell. 10% of the randomly selected reflections were used for calculating *R*_free_ values.

	Flip-holder	NAM-based holder (Lee *et al.*, 2019 ▸)	Kapton film holder (Li *et al.*, 2018 ▸)
Data collection			
X-ray source	BL11C, PAL	BL11C, PAL	BL17U1, SSRF
Exposure time (ms)	100 ms	100 ms	Not available
Beam size (µm)	8.5 (horizontal) × 4.1 (vertical)	8.5 (horizontal) × 4.1 (vertical)	67 (horizontal) × 23 (vertical)
Wavelength (Å)	0.97942	0.97942	0.97942
Incident flux (photons s^−1^)	∼1.3 × 10^12^	∼1.3 × 10^12^	∼3.8 × 10^12^
No. of crystals	∼170 crystals µl^−1^, 30 µl	∼2100 crystals µl^−1^, 20 µl	N/A
No. of collected images	19600	56700	20 (set) × 390
No. of integrated images	5098	41916	N/A
No. of indexed images	3806	21670	N/A

Scaling			
Space group	*P*4_3_2_1_2		
Unit-cell dimensions			
*a*, *b*, *c* (Å)	78.90, 79.17, 37.39	79.45 79.45 38.45	78.9, 78.9, 37.1
α, β, γ (°)	90.0, 90.0, 89.74	90, 90, 90	90, 90, 90
Resolution (Å)	25.7–1.89 (1.94–1.89)	80.0–1.50 (1.55–1.50)	55.8–1.34
No. of unique reflections	17738	20320	27073
*R*_split_ (%)†	16.65 (31.91)	8.97 (50.97)	N/A
*I*/σ(*I*)	4.8 (3.2)	7.94 (2.24)	27.89 (3.55)
CC_1/2_	0.96 (0.71)	0.98 (0.58)	N/A
CC*	0.99 (0.91)	0.99 (0.85)	N/A
Multiplicity	146.75 (106.3)	427.0 (296.0)	19.8 (2.8)
Completeness (%)	100 (100.00)	100.00 (100.00)	98.8 (95.4)

Refinement			
Resolution (Å)	25.7–1.89	56.18–1.50	N/A
*R*_work_ / *R*_free_ (%)	22.81 / 24.57	16.66 / 18.53	18.0 / 20.0
No. atoms protein / water	1001 / 44	1001 / 83	1108 / 107
RMSD bond lengths (Å) / angles (°)	0.003 / 0.510	0.014 / 1.703	0.02 / 1.9
Ramachandran plot (%) favored / allowed / outliers	98.43 / 1.57 / 0	99.21 / 0.79 / 0	96.85 / 3.15 / 0

† *R*_split_ = 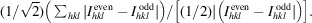

## Data Availability

Data available within the article or its supplementary materials.
