# Supplementary material for: Novel fixed-target serial crystallography flip-holder for macromolecular crystallography beamlines at synchrotron radiation sources
Source: J Synchrotron Radiat. 2025 Feb 3;32(Pt 2):315–20. doi: 10.1107/S1600577524011664 (PMC11892908; doi:10.1107/S1600577524011664)
Supplement: Supplementary file 2 [file s-32-00315-sup2.pdf]

JOURNAL OF SYNCHROTRON RADIATION

Volume 32 (2025)

Supporting information for article:

Novel fixed-target serial crystallography flip-holder for macromolecular crystallography beamlines at synchrotron radiation sources

Do-Heon Gu, Dong Tak Jeong, Cheolsoo Eo, Pil-Won Seo, Jeong-Sun Kim and Suk-Youl Park

(a) (b)

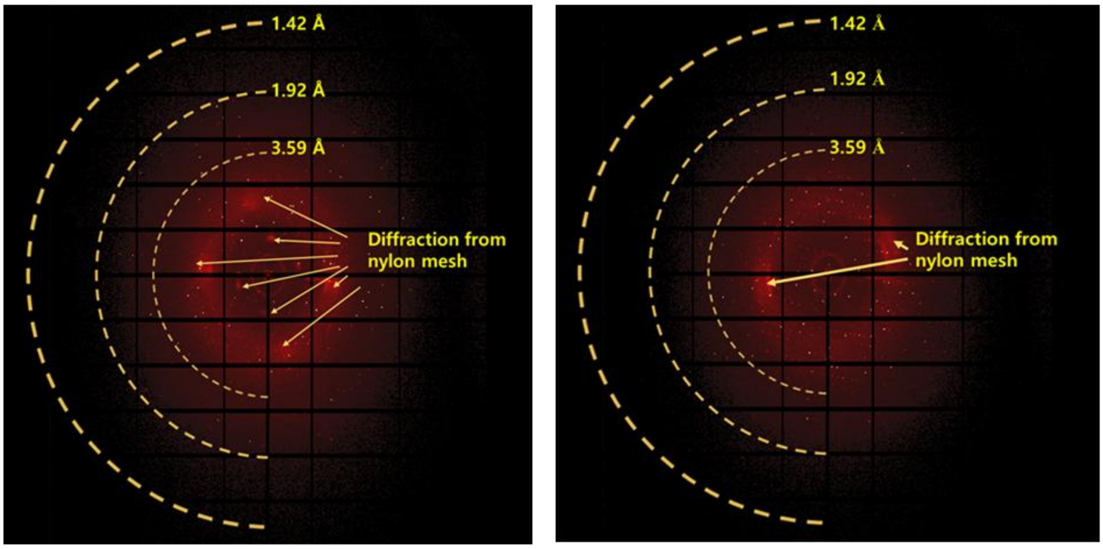

Figure S1 Diffraction images of the nylon mesh and lysozyme crystal. Yellow dashed lines indicate the resolution range and yellow arrow indicates the diffraction from the nylon mesh. (a) The first diffraction pattern image of the nylon mesh. (b) The second diffraction pattern image.
